# *Ultrabithorax* confers spatial identity in a context-specific manner in the *Drosophila* postembryonic ventral nervous system

**DOI:** 10.1186/1749-8104-7-31

**Published:** 2012-09-11

**Authors:** Elizabeth C Marin, Katie E Dry, Danielle R Alaimo, Kirstin T Rudd, Anthony R Cillo, Michael E Clenshaw, Nicolas Negre, Kevin P White, James W Truman

**Affiliations:** 1 Biology Department, Bucknell University, Lewisburg, PA, USA; 2 Neuroscience Program, Bucknell University, Lewisburg, PA, USA; 3 Cell Biology/Biochemistry Program, Bucknell University, Lewisburg, PA, USA; 4 Institute for Genomics & Systems Biology, University of Chicago, Chicago, IL, USA; 5 Janelia Farm Research Campus, Howard Hughes Medical Institute, Ashburn, VA, USA

**Keywords:** Hox, Programmed cell death, CNS, Neuroblast lineages

## Abstract

**Background:**

In holometabolous insects such as *Drosophila melanogaster*, neuroblasts produce an initial population of diverse neurons during embryogenesis and a much larger set of adult-specific neurons during larval life. In the ventral CNS, many of these secondary neuronal lineages differ significantly from one body segment to another, suggesting a role for anteroposterior patterning genes.

**Results:**

Here we systematically characterize the expression pattern and function of the Hox gene *Ultrabithorax* (*Ubx*) in all 25 postembryonic lineages. We find that *Ubx* is expressed in a segment-, lineage-, and hemilineage-specific manner in the thoracic and anterior abdominal segments. When *Ubx* is removed from neuroblasts via mitotic recombination, neurons in these segments exhibit the morphologies and survival patterns of their anterior thoracic counterparts. Conversely, when *Ubx* is ectopically expressed in anterior thoracic segments, neurons exhibit complementary posterior transformation phenotypes.

**Conclusion:**

Our findings demonstrate that *Ubx* plays a critical role in conferring segment-appropriate morphology and survival on individual neurons in the adult-specific ventral CNS. Moreover, while always conferring spatial identity in some sense, *Ubx* has been co-opted during evolution for distinct and even opposite functions in different neuronal hemilineages.

## Background

The insect ventral CNS, like the body as a whole, is built on a plan of repeating segmental units that then undergo regional specialization. The neurons of a segmental unit arise from a stereotyped two-dimensional array of 30 uniquely identifiable neural stem cells (neuroblasts, NB) per hemisegment [1-3]. These NBs undergo repeated asymmetric divisions, thereby producing a series of ganglion mother cells, GMCs [4], each of which divides to produce a pair of postmitotic daughters [5,6]. These daughters then acquire distinct fates via Notch signaling [7,8]. In insects with complete metamorphosis, like *Drosophila melanogaster*, the NBs typically have an initial burst of proliferation to generate the neurons of the larval CNS and then later a subset feature an extended proliferative period during larval life, producing most of the neurons of the adult CNS [9,10]. During the postembryonic neurogenic phase, Notch signaling between sibling cells produces two morphologically distinguishable cell types that accumulate to form two distinct hemilineages, one of which may be eliminated by programmed cell death [10].

In the embryo, the NB arrays are almost identical between thoracic and abdominal neuromeres [11], although there are some regional differences in the neurons produced by thoracic versus abdominal homologs [12-14]. During the postembryonic neurogenic phase, however, there are dramatic differences between the numbers of thoracic versus abdominal NBs [15]. Within the thorax particular lineages exhibit segment-specific differences in their cellular composition [10].

Given their roles in anteroposterior patterning of the embryonic CNS (reviewed in [16]), the Hox genes are excellent candidates for conferring segmental identity in the postembryonic nervous system. For example, in late stage embryos, Abdominal-A (Abd-A) represses proliferation of many NBs in the abdomen [17], and a burst of *abd-A* expression causes the apoptosis of persistent abdominal lineages during the third instar [17,18]. Also, Ultrabithorax (Ubx) represses the formation of leg neuropils in the first abdominal segment (A1) [19], and in *Ubx*^*-*^ animals, thoracic-specific NBs are retained in the A1 neuromere during the postembryonic neurogenic period [20].

The development of methods to label and manipulate NB lineages [21] has allowed the detailed characterization of the postembryonic lineages that generate the adult-specific neurons [9,10]. Using these methods we find that *Ubx* is expressed in a segment-, lineage-, and sibling-specific manner that correlates with morphological differences observed in different segments for particular lineages. Moreover, removal of *Ubx* from a lineage via the MARCM (mosaic analysis with a repressible cell marker) method results in anterior transformation of its morphology and survival pattern, whereas ectopic expression of *Ubx* results in posterior transformation. Interestingly, *Ubx* can promote survival, death, and/or segment-specific changes in neurite morphology, depending on the hemilineage. Taken together, these data demonstrate that *Ubx* has been co-opted during evolution to regulate the segmental identity of secondary neurons in a context-dependent manner during development.

## Results

### Overview of *Ubx* expression in the larval nervous system

**Figure 1:**
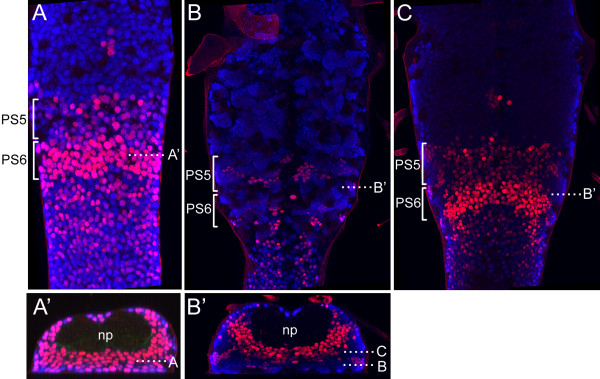
**Expression of Ubx in the ventral CNS of first instar and late third instar larvae.** (**A**) Dorsal projection showing Ubx expression (red) in parasegments (PS) 5 and 6. (**A**’) Transverse projection at the indicated level in PS6. (**B**,**C**) Optical sections at the level of the adult-specific lineages (**B**) and of the larval neurons (**C**). (**B**’) Transverse projection showing the level of the optical sections and the uniform expression in the larval neuron layer around the neuropil (np) and the variable staining of the adult-specific lineages. Blue: elav; np: neuropil; PS: parasegment; Ubx: Ultrabithorax.

As initially described by White and Wilcox [22], the major domain of Ubx expression in the embryonic CNS is parasegment 6 (Figure 1A), with weaker expression in parasegment 5 (posterior T2 and anterior T3) and an isolated cluster of neurons in the midline of parasegment 4. Posterior to A1, Ubx expression is weak and spotty but still occurs in some neurons through A7. Within parasegment 6, the great majority of the neurons show strong Ubx expression (Figure 1A’).

**Figure 2:**
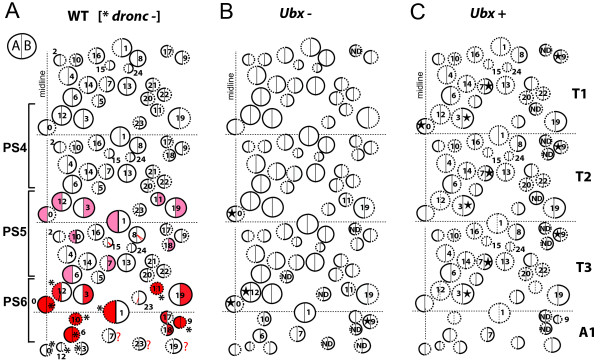
**Summary of expression of Ultrabithorax and effects of Ultrabithorax manipulation in the ventral adult-specific lineages.** Circles show the relative size and position of the segmental lineages. The two halves of each circle represent the A (Notch^ON^) and B (Notch^OFF^) hemilineages. Neurons in hemilineages with dashed borders die soon after their birth. **(A)** Summary of expression of Ubx in wild-type (WT) clones and in clones in which cell death is blocked by mutation of the *dronc* gene. Ubx expression typically showed a hemilineage restriction and was weak to moderate (pink) or strong (red) depending on parasegment (PS). The Ubx expression in hemilineages that normally died was revealed in *dronc*^*-*^ clones (*). Red ? = no Ubx expression data. **(B**,**C)** The effects of loss of Ubx (Ubx-) or ectopic expression of Ubx (Ubx+) in MARCM clones. Only the lineages that were changed by a given treatment are numbered. The changes include the survival or death of hemilineages (solid versus dashed outlines) and alteration in projection patterns (Star). A1: first abdominal segment; ND: no data; PS4-6: fourth to sixth parasegments; T1-T3: first to third thoracic segments; Ubx: Ultrabithorax; WT: wild-type.

By the end of the last (third) larval stage, the larval neurons have been joined by clusters of secondary neurons. The former are in a compact layer next to the neuropil, whereas the latter are in superficial clusters that extend from the larval neuron layer to the surface of the CNS. The larval neurons show the same pattern of Ubx expression as seen at hatching (Figure 1B’, C). Ubx expression in the postembryonic lineages is also mostly confined to parasegments 5 and 6, with that expression in the latter being stronger. However, unlike in the larval neurons, Ubx expression in the clusters of postembryonic-born neurons was quite heterogeneous, even in parasegment 6. The NBs and GMCs did not express Ubx, but Ubx expression within the associated cluster of postembryonic neurons varied from cluster to cluster (Figure 1B,B’), suggesting a lineage-based regulation. The T2 lineages that exhibit any Ubx expression are 0, 3, 11, 12, and 19, all of which are in the *engrailed* domain (JWT & D.W. Williams, unpublished work) and, thus, in the anterior portion of parasegment 5. These lineages express much higher levels of Ubx in T3 but fail to express it in A1 (parasegment 7). Ubx expression in the postembryonic lineages is summarized in Figure 2, and examples of expression patterns for the positive lineages are given in the following figures.

Lineages that were Ubx positive typically had all Ubx+ cells or roughly equal numbers of Ubx+ and Ubx- neurons. For the latter cases, our Ubx manipulations described below argue that one sibling from the GMC division becomes Ubx+ and the other Ubx-, thereby resulting in roughly equal numbers of the two expression types. There were a few lineages in which Ubx expression appeared not to be divided along hemilineage lines. Lineage 12 in segment T3 and lineage 1 in A1 both had a few Ubx+ cells apically, near the NB and GMCs. However, as shown below, in both lineages Ubx expression is responsible for the death of the neurons of one hemilineage, and the cells that we observed were the newly-born cells that had not yet initiated programmed cell death. Expression patterns that were clearly not hemilineage related were seen for the largely negative lineages 8, 15, and 23. Each had one to a few weakly Ubx+ neurons in T3, but our Ubx manipulations did not reveal a role for this expression.

### *Ubx* regulates segment-specific neuronal programmed cell death of particular hemilineages

#### Lineage 1

**Figure 3:**
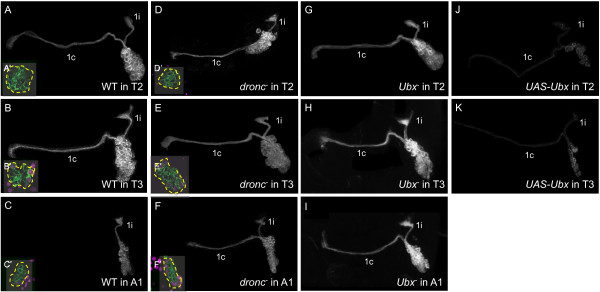
**Dorsal view of neuroblast clones showing Ultrabithorax expression and genetic manipulation in lineage 1.** Insets: Ubx expression (magenta) in a mid-clone optical section. **(A**-**C)** Wild-type clones. (**C**) Bundle 1c is absent in A1. **(D**-**F)***dronc*^*-*^ clones. (**F**) Bundle 1c is present in A1. **(G**-**I)***Ubx*^*-*^ clones. (**I**) Bundle 1c is present in A1. **(J**-**K)***UAS-Ubx* clones. Bundles 1i and 1c are very thin and lack elaboration. White or green, anti-CD8. Ubx: Ultrabithorax.

Lineage 1 provides a striking example of segment-specific survival in the secondary lineages. The neurons in the 1A hemilineage form the contralateral (1c) axon bundle that projects across the anterior ventral (aV) commissure to the contralateral leg neuropil, and those in the 1B hemilineage form the ipsilateral (1i) bundle that projects to the next anterior leg neuropil [10]. Both hemilineages are present in segments T2 (n = 13/13, 1c axon bundle diameter = 2.97 ±0.60 μm, Figure 3A) and T3 (n = 12/12, 3.06 ±0.37 μm, Figure 3B). However, in T1, the 1B hemilineage that would have projected to S3 is absent [9] (n = 7/9, not shown) or truncated (n = 2/9, not shown), while in A1, the contralaterally projecting 1A hemilineage is missing [9] (n = 4/9, Figure 3C) or exhibits only a few aborted axons (n = 5/9, not shown), resulting in a greatly reduced axon bundle with a diameter of only 0.83 ±0.91 μm. The absent or reduced hemilineages correlate with the absence of leg neuropils in S3 and A1.

We examined the Ubx expression pattern in wild-type (WT) and *dronc*^*-*^ clones. In WT clones in T1 (n = 3/3, data not shown) and T2 (n = 7/7, Figure 3A’), all lineage 1 cells were Ubx-, while approximately half of the cells in T3 clones were weakly positive for Ubx (n = 3/3, Figure 3B’). In A1, all or most cells were Ubx-, with a few cells near the NB occasionally observed to be strongly positive (n = 3/5, Figure 3C’). For *dronc*^*-*^ clones, in which programmed cell death was blocked, the A1 cluster was enlarged and showed a robust 1c bundle consistent with the survival of the 1A sibling (n = 11/12, 2.91 ± 0.56 μm, Figure 3F). Approximately half of the cells in the enlarged cluster were strongly positive for Ubx (n = 9/9, Figure 3F’), suggesting that the 1A sibling in A1 expresses a high level of Ubx prior to dying.

Loss and gain of function experiments confirmed that *Ubx* regulates the survival of lineage 1A neurons. *Ubx*^*-*^ clones in A1 exhibited a robust 1c bundle as well as the expected 1i bundle (n = 23/24, 3.53 ± 0.96 μm, Figure 3I). Interestingly, the ectopic 1c bundle hooked upwards towards T3, rather than taking the expected trajectory towards the posterior part of the segment. A similar phenotype was also seen in *dronc-* clones for lineage 1 in segment A1. This altered pathway may be due to a lack of leg neuropil target cues in abdominal segments. The misexpression of *Ubx* in *UAS-Ubx* clones apparently led to the death of both hemilineages of lineage 1 neurons, regardless of segment. Only a few thoracic lineage 1 clones were observed, and those had few cells and very thin, faint projections, most likely indicating that the neurons were dying (n = 11/12, axon bundle diameter = 1.80 ± 0.27 μm, Figure 3J, K). Thus, a high level of Ubx expression can result in the death of the neurons of both hemilineages, although only hemilineage 1A neurons normally express it. Also, although the hemilineage 1A neurons in T3 normally express a moderate level of Ubx, they die in response to the high levels in the MARCM clones. Therefore, the ability of Ubx to cause the death of these neurons appears to be concentration dependent.

#### Lineage 6

**Figure 4:**
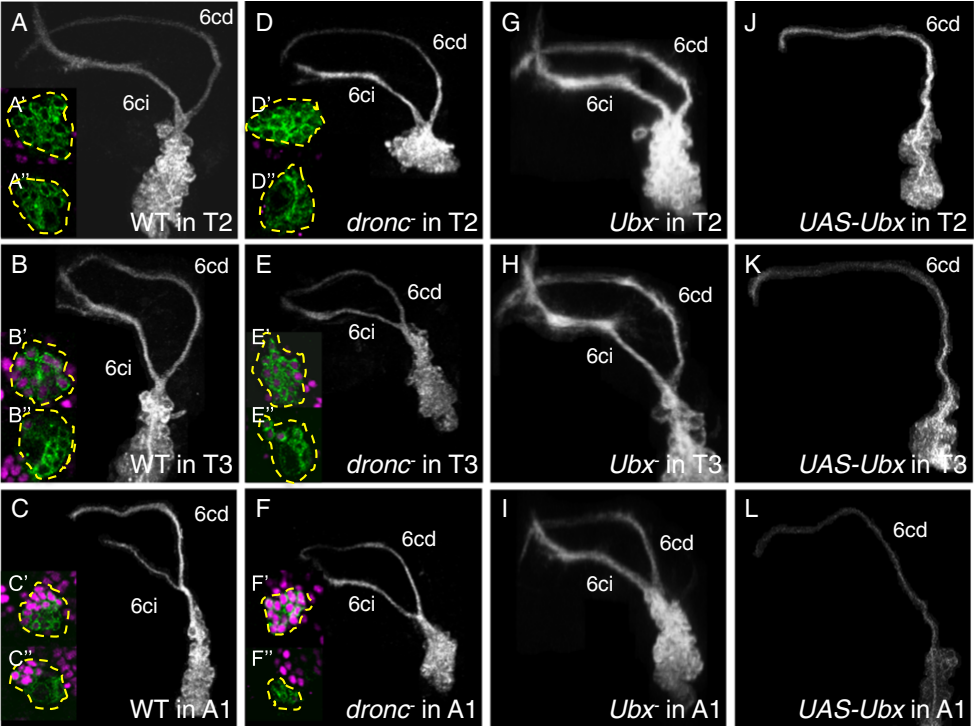
**Dorsal view of neuroblast clones showing Ultrabithorax expression and genetic manipulation in lineage 6.** Insets: Ubx expression (magenta) at mid-clone (upper) or next to neuroblast (lower). **(A-C)** Wild-type clones. (**C**) Bundle 6ci is thin and truncated in A1. **(D-F)***dronc*^*-*^ clones. (**F**) Bundle 6ci is robust in A1. **(G-I)***Ubx*^*-*^ clones. (**I**) Bundle 6ci is robust in A1. **(J-L)***UAS-Ubx* clones. Bundle 6ci is missing in all segments. White or green, anti-CD8. Ubx: Ultrabithorax.

NB 6 produces two hemilineages: 6A, whose axon bundle 6cd projects to the posterior dorsal (pD) commissure, and 6B, whose axon bundle 6ci projects along the posterior intermediate (pI) commissure before turning anteriorly [10] (T2: n = 13/13, 6ci axon bundle diameter = 4.86 ±1.16 μm, Figure 4A; T3: n = 6/6, 4.63 ±1.11 μm, Figure 4B). In segment A1, the 6ci bundle is significantly reduced and/or truncated [9] (n = 5/6, 0.63 ±0.99 μm, Figure 4C), suggesting decreased survival of the 6B sibling. This was confirmed by *dronc*^*-*^ lineage 6 clones in A1 that featured robust 6ci bundles projecting from an enlarged cell body cluster (n = 9/9, 2.96 ±0.68 μm, Figure 4F).

WT lineage 6 clones lacked Ubx expression in T1 (n = 12/12, not shown) and T2 (n = 14/14, Figure 4A’, A”), were weakly Ubx+ in T3 (n = 12/22, Figure 4B’,B”), and were strongly Ubx+ in A1 except for the youngest cells (n = 15/15, Figure 4C’,C”). *dronc*^*-*^ clones in A1 also featured strong Ubx expression in all but the youngest cells (n = 7/7, Figure 4F’,F”), implying that most or all of the 6B siblings that typically die in A1 also express high levels of Ubx.

In comparison with WT lineage 6 clones in A1, *Ubx*^*-*^ clones were larger and exhibited a robust 6ci bundle (n = 7/7, 4.26 ± 0.74 μm, Figure 4I), indicating the survival of the 6B hemilineage. Conversely, with ectopic expression of *Ubx* in lineage 6, the 6ci bundle was much reduced or absent in all segments (T2: n = 16/17, 0.41 ± 0.77 μm, Figure 4J; T3: n = 22/22, 0.49 ± 0.97 μm, Figure 4K; A1: n = 16/16, 0 ± 0 μm, Figure 4L), although the 6 cd bundle was present throughout. These results show that a high level of Ubx expression is both necessary and sufficient for death of the 6B but not the 6A hemilineage.

#### Lineage 19

**Figure 5:**
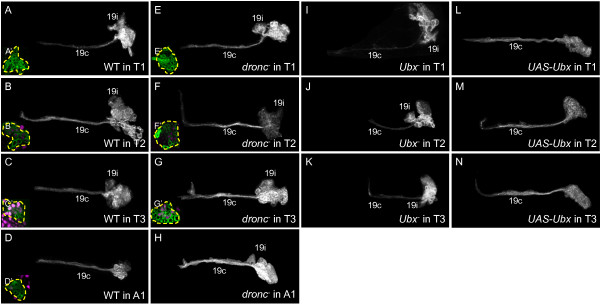
**Dorsal view of neuroblast clones showing Ultrabithorax expression and genetic manipulation in lineage 19.** Insets: Ubx expression (magenta) at mid-clone. **(A-D)** Wild-type clones. (**A**) Bundle 19c is thin and truncated in T1. (**D**) Bundle 19i is absent in A1. **(E-H)***dronc*^*-*^ clones. (**E**) Bundle 19c is robust in T1. (**H**) Bundle 19i is present but lacks elaboration in A1. **(I-K)***Ubx*^*-*^ clones. Bundle 19c is very thin in all segments. **(L-N)***UAS-Ubx* clones. Bundle 19i is missing in all segments. White or green, anti-CD8. Ubx: Ultrabithorax.

In the thorax, the neurons of the 19A hemilineage projects diffusely into the ipsilateral leg neuropil via bundle 19i, whereas those of the 19B hemilineage form bundle 19c, which projects contralaterally in the pI commissure [10]. Both hemilineages are found in T2 (n = 5/5, 19c axon bundle diameter 4.52 ± 1.42 μm, Figure 5B) and T3 (n = 8/8, 4.15 ± 0.38 μm, Figure 5C), but in T1 there are only a few contralaterally projecting 19B cells, resulting in a greatly reduced 19c bundle (n = 3/3, 0.61 ± 1.06 μm, Figure 5A) [9]. In A1, by contrast, the 19B cells are present and form a robust 19c bundle, but the 19A cells are absent (n = 1/1, Figure 5D). Ubx expression was absent from WT T1 clones (n = 12/12, Figure 5A’). About half of the cells in T2 clones showed weak Ubx expression (n = 10/12, Figure 5B’), and about half of the cells in T3 clones were strongly Ubx+ (n = 15/15, Figure 5C’). Cells contributing to 19c in the A1 lineage 19 clones were Ubx- (n = 1/1, Figure 5D’).

In *dronc*^*-*^ T1 clones, both the 19A and 19B hemilineages were present, judging from the increased thickness of the 19c bundle (n = 10/10, 4.23 ± 0.75 μm, Figure 5E), but there still was no Ubx expression (n = 7/7, Figure 5E’), indicating that the 19B neurons are normally Ubx- in that segment. *dronc*^*-*^ clones in T2 (n = 7/7, 19c axon bundle diameter 4.36 ± 0.24 μm, Figure 5F; n = 3/4, Figure 5F’) and T3 (n = 10/10, 4.03 ± 0.51 μm, Figure 5G; n = 7/7, Figure 5G’) exhibited morphologies and Ubx expression patterns that were identical to those of WT. In segment A1, *dronc*^-^ clones were enlarged and showed a 19i projection diagnostic of the survival of 19A neurons (n = 1, Figure 5H). However, no A1 *dronc*^*-*^ clones counterstained for Ubx expression were recovered.

With the loss of *Ubx,* lineage 19 clones in T2 (n = 6/6, axon bundle diameter 1.69 ± 1.47 μm, Figure 5J) and T3 (n = 8/8, 1.39 ± 1.49 μm, Figure 5K) had few or no axons in the 19c bundle, suggesting that most of the 19B neurons had died, and that *Ubx* is required for the survival of the thoracic 19B neurons. Accordingly, overexpressing *Ubx* resulted in a robust 19c bundle in T1 (n = 9/9, 4.80 ± 0.59 μm, Figure 5L) as well as T2 (n = 19/19, 4.90 ± 0.77 μm, Figure 5M) and T3 (n = 7/7, 5.49 ± 0.56 μm, Figure 5N), but the 19A neurons that produce the 19i bundle were missing from all three thoracic segments (n = 30/30). Surprisingly, *Ubx* is both necessary and sufficient for the *survival* of the 19B siblings in the thorax, but can cause *death* of the 19A siblings. No *Ubx*^*-*^ or *UAS-Ubx* clones were recovered in A1 (parasegment 7), where the fates of lineage 19 neurons may be out of the domain of Ubx action.

### *Ubx* confers segment-specific projections on particular hemilineages

#### Lineage 0

**Figure 6:**
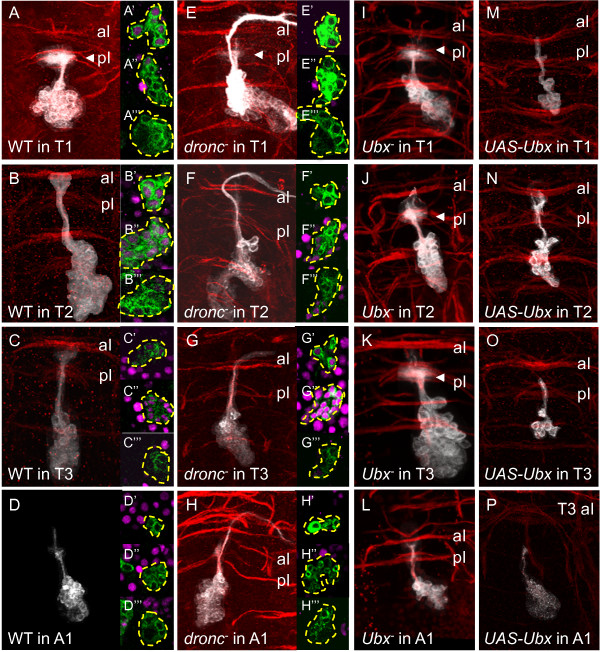
**Dorsal view of neuroblast clones showing Ultrabithorax expression and genetic manipulation in lineage 0.** Insets: Ubx expression (magenta) at basal (upper), middle (middle), or apical (lower) parts of clone. **(A-D)** Wild-type clones. (**A**) Bundle 0im terminates and elaborates in the pI in T1 (arrowhead). **(E-H)***dronc*^*-*^ clones. A second axon bundle extending past the aI and turning laterally appears in all segments. (**E**) Bundle 0im terminates and elaborates in the pI in T1 (arrowhead). **(I-L)***Ubx*^*-*^ clones. Bundle 0im terminates and elaborates in the pI in T2 (**J**) and T3 (**K**). **(M-P)***UAS-Ubx* clones. (**M**) Bundle 0im terminates in the aI in T1. White or green = anti-CD8; red = anti-neurotactin. aI: anterior intermediate commissure; pI: posterior intermediate commissure; Ubx: Ultrabithorax.

In lineage 0 clones in T1, a single interneuron axon bundle from the 0A sibling [10] projects anterodorsally along the midline and terminates diffusely on the commissure (n = 4/4, Figure 6A). However, in T2 (n = 4/5, Figure 6B), T3 (n = 3/3, Figure 6C), and A1 (n = 4/4, Figure 6D), the 0A bundle extends further to the anterior intermediate commissure (aI) [9]. We see the same segment-specific targeting of these interneurons in *dronc*^-^ clones (T1: n = 6/6, Figure 6E; T2: n = 9/10, Figure 6F; T3: n = 2/5, Figure 6G; A1: n = 5/5, Figure 6H), but the local interneurons are now joined by the surviving 0B projection neurons.

The distinct targets of the 0A neurons are correlated with differences in their expression of Ubx. WT cell clusters in T1 (n = 10/11, Figure 6A’-A”’) were Ubx- except for the oldest (possibly embryonic born) cells, while those in T2 exhibited moderate levels of Ubx in older cells (n = 4/7, Figure 6B’-B”’) and those in T3, high levels of Ubx in all but the oldest and youngest cells (n = 10/10, Figure 6C’-C”’). WT clones in A1 (parasegment 7) were negative for Ubx expression (n = 4/4, Figure 6D’-D”’). Similar Ubx expression patterns were seen in *dronc*^*-*^ clones in all four segments (T1, n = 3/4, Figure 6E’-E”’; T2, n = 4/6, Figure 6F’-F”’; n = 5/5, Figure 6G’-G”’; A1, n = 4/5, Figure 6H’-H”’).

In *Ubx*^*-*^ clones, the 0A interneurons in T2 (n = 5/5, Figure 6J) and T3 (n = 7/7, Figure 6K) adopted a T1-like morphology, projecting diffusely over the pI commissure. Conversely, with ectopic expression of Ubx, the 0A interneurons in T1 adopted a posterior morphology, now projecting to the aI commissure (n = 13/15, Figure 6M). Taken together, these data demonstrate that *Ubx* acts to specify the segment-appropriate axon morphology of lineage 0 neurons in the thoracic neuromeres. Consistent with their lack of Ubx expression in WT or *dronc*^*-*^ clones, the axon morphology of A1 clones were unaffected by Ubx manipulation (n = 3/3, Figure 6L).

#### Lineage 9

**Figure 7:**
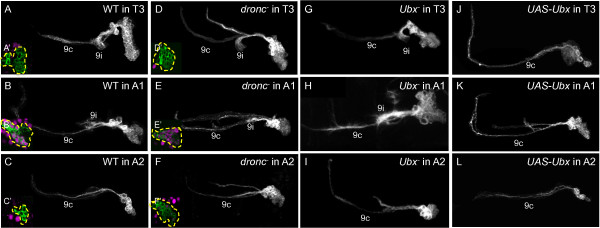
**Dorsal view of neuroblast clones showing Ultrabithorax expression and genetic manipulation in lineage 9.** Insets: Ubx expression (magenta) at mid-clone. **(A-C)** Wild-type clones. (**A**) Bundle 9i is robust and curls around leg neuropil in T3. (**B**) Bundle 9i is reduced and travels adjacent to 9c in A1. (**C**) Bundle 9i is reduced and travels adjacent to bundle 9c in A2. **(D-F)***dronc*^*-*^ clones. A second axon bundle appears in all segments. Bundle 9i follows same pattern as in wild-type. **(G-I)***Ubx*^*-*^ clones. (**H**) Bundle 9i is robust and curls around leg neuropil in A1. **(J-L)***UAS-Ubx* clones. Bundle 9i is missing or reduced and travels adjacent to bundle 9c in all segments. White or green, anti-CD8. Ubx: Ultrabithorax.

In the thorax, lineage 9 exhibits a robust ipsilateral projection (9i) that curls around the leg neuropil and a thin contralateral projection (9c) that crosses the midline in the aV commissure [9] (n = 4/4, Figure 7A). However, both of these projections are generated by the neurons of hemilineage 9A [10], while neurons from the 9B hemilineage typically die but survive in *dronc*^*-*^ clones to generate a more dorsal contralateral projection (bundle 9ic) (n = 9/11, Figure 7D). In A1, the 9A hemilineage is smaller, with a 9i projection that is less pronounced and travels a short distance with bundle 9c (n = 4/5, Figure 7B). *dronc*^*-*^ clones in A1 are similar to WT except they have the dorsal 9ic bundle (n = 3/4, Figure 7E). In segment A2, the 9i bundle is no longer distinguishable from 9c in either WT (n = 4/5, Figure 7C) or *dronc*^*-*^ clones (n = 3/3, Figure 7F).

WT lineage 9 clones in T3 did not express Ubx (n = 10/10, Figure 7A’), but most cells of those in A1 were strongly Ubx+ (n = 8/9, Figure 7B’), while those in A2 were Ubx- (n = 8/8, Figure 7C’). *dronc*^*-*^ clones in T3 were Ubx- (n = 3/3, Figure 7D’), while those in A1 featured strong Ubx expression (n = 4/4, Figure 7E’) and those in A2 were mixed Ubx + and Ubx- (n = 2/2, Figure 7F’).

*Ubx*^*-*^ clones in T3 looked normal (n = 9/13, Figure 7G), but those in A1 were larger and exhibited a much more robust and diffuse 9i process as compared with WT (n = 4/5, Figure 7H). *Ubx*^*-*^ clones in A2 either featured more robust 9i projections traveling along with 9c (n = 8/14) or an additional, more dorsal contralateral projection similar to those seen in *dronc*^*-*^ clones (n = 7/14, Figure 7I). For thoracic *UAS-Ubx* MARCM clones, the 9i bundle was either absent (n = 2/15 for T3, not shown) or remained closely associated with the 9c bundle and lacked the characteristic medial “hook” (n = 11/15 for T3, Figure 7J). These results suggest that for lineage 9, *Ubx* regulates both cell survival and axon guidance in A1 and A2.

#### Lineage 12

**Figure 8:**
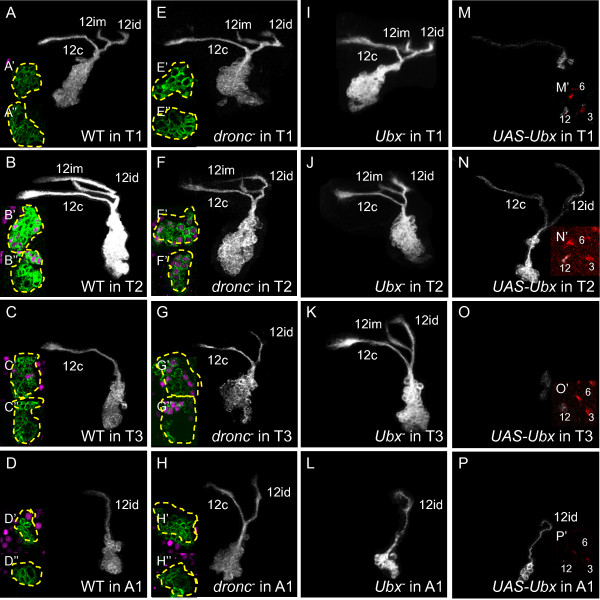
**Dorsal view of neuroblast clones showing Ultrabithorax expression and genetic manipulation in lineage 12.** Insets show either Ubx immunostaining (magenta) of sections at mid-clone (**A**’-**H**’) or adjacent to the neuroblast (**A**”-**H**”); or neurotactin immunostaining (red): single confocal slices shortly after the axon bundle enters the neurotactin scaffold (**M**’-**P**’). (**A-D**) Wild-type clones. (**C**) Only bundle 12c is present in T3. (**D**) Only 12id is present in A1. (**E-H**) *dronc*^*-*^ clones. (**G**) Bundles 12c and 12id, but not 12im, are present in T3. (**H**) Bundles 12c and 12id, but not 12im, are present in A1. (**I-L**) *Ubx*^*-*^ clones. (**K**) Bundles 12im, id, and c are present in T3. (**M-P**) *UAS-Ubx* clones. Cell bodies are missing and axon bundles reduced or missing in all segments. (**M’-P’**) Lineage 12 is still identifiable based on characteristic position in neurotactin scaffold. White or green, anti-CD8, Ubx: Ultrabithorax.

For lineage 12, the 12A neurons form the 12i bundle to presumed flight neuropil, and the 12B neurons form the 12c bundle that extends to the contralateral leg neuropil [10]. Both hemilineages are present in T1 (n = 6/6, Figure 8A) and T2 (n = 4/4, Figure 8B), although in T1 the 12i bundle always exhibits a dorsal bifurcation, 12im [9], whereas in T2 it may or may not be branched depending on genetic background (data not shown). In T3, the 12A hemilineage producing the 12i bundle is absent (n = 3/5) or greatly reduced (n = 2/5) [9] (Figure 8C), while in A1 the 12B hemilineage producing the 12c bundle is absent (n = 2/2, Figure 8D).

WT T1 clones were negative for Ubx (n = 12/12, Figure 8A’,A”). T2 clones were approximately half negative and half weakly positive for Ubx (n = 8/11, Figure 8B’,B”), while T3 clones were mostly negative except for a few strongly positive cells near the NB (n = 19/21, Figure 8C’,C”). In *dronc*^*-*^ clones in T3, both the 12A and 12B hemilineages were present, producing robust 12i and 12c axon bundles (n = 7/7, Figure 8G), and the clusters were approximately half Ubx- and half strongly Ubx+ (n = 4/4, Figure 8G’,G”). Thus, the few apical Ubx+ cells we observed in WT clones were likely newly born 12A neurons prior to their death. Interestingly, the 12im bifurcation was absent from all *dronc*^*-*^ clones in T3 as well as those in A1 (n = 7/7, Figure 8H).

*Ubx*^*-*^ clones in T3 featured surviving 12A neurons, which made a bifurcated 12im bundle (n = 9/10, Figure 8K). This phenotype resembled that of WT lineage 12 clones in T1 rather than that of *dronc*^*-*^ clones in T3, indicating that *Ubx* confers segment-appropriate axon projection patterns of the 12A neurons as well as regulating their death. In segment A1 (parasegment 7), by contrast, loss of *Ubx* had no apparent effect (n = 7/7, Figure 8L), consistent with the absence of Ubx expression in both wild-type (n = 16/16, Figure 8D’,D”) and *dronc*^*-*^ (n = 8/8, Figure 8H’,H”) A1 clones.

Misexpression of *Ubx* caused the death of both 12A and 12B siblings in all three thoracic segments, as evidenced by dramatically thinned and/or absent projections (T1: n = 17/17, Figure 8M; T2: n = 4/4, Figure 8N; T3: n = 8/8, Figure 8O) and no 12i bifurcation. The characteristic position of the lineage 12 bundle in the neurotactin scaffold relative to lineages 3 and 6 permitted unequivocal identification of this bundle even in the absence of CD8-GFP-labeled processes (Figures 8M’, N’, O’). Where the clone should reside, we often saw a small cluster of cells with truncated or no projections. In such preparations the neurotactin-positive bundles (12i and 12c) were missing, confirming that these cells did not survive. These data strongly suggest that a high level of Ubx expression promotes the death of both lineage 12 siblings, while an intermediate level of Ubx permits survival but controls the segment-specific bifurcation of the 12i bundle.

In addition to these three examples, we also found a possible role for *Ubx* in determining the axon projections of lineages 3 and 7 (Figure 2). Overexpression of *Ubx* in lineage 3 resulted in survival of only the 3id sibling in the thoracic segments (n = 28/43), and the terminal elaborations normally found in T1 were missing (n = 43/43). Overexpression of *Ubx* in lineage 7 resulted in the axon bundle turning posteriorly instead of anteriorly (n = 16/51) or failing to turn (n = 32/51) after crossing the midline in all three thoracic segments (data not shown). However, given that there was no abnormal phenotype in *Ubx*^*-*^ clones for either lineage, we cannot conclude definitively that *Ubx* normally regulates axon guidance in these lineages.

### *Ubx* promotes the programmed cell death of entire lineages

#### Lineage 10

**Figure 9:**
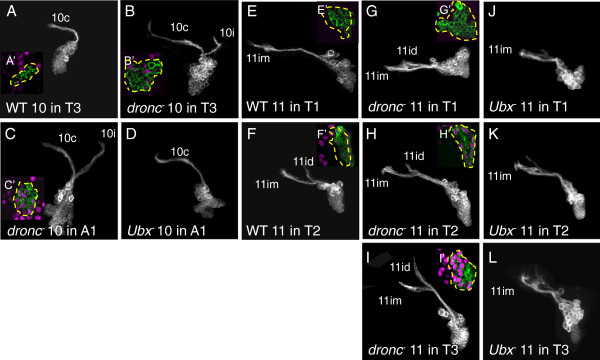
**Dorsal view of neuroblast clones showing Ultrabithorax expression and genetic manipulation in lineages 10 and 11.** Insets: Ubx immunostaining (magenta) at mid-clone. (**A**) Wild-type lineage 10 clone in T3. Only bundle 10c is present. (**B, C**) *dronc*^*-*^ lineage 10 clones. Bundles 10c and 10i are both present in all segments. (**D**) *Ubx*^*-*^ lineage 10 clone in A1. Only bundle 10c is present. (**E-F**) Wild-type lineage 11 clones. (**E**) Only bundle 11im is present in T1. (**F**) Bundles 11im and 11id are both present in T2. (**G-I**) *dronc*^*-*^ lineage 11 clones. Bundles 11im and 11id are both present in all thoracic segments. (**J-L**) *Ubx*^*-*^ lineage 11 clones. Bundle 11im, but not 11id, is present in all thoracic segments. White or green, anti-CD8, Ubx: Ultrabithorax.

Lineage 10 is found only in the three thoracic segments and is represented there by only the 10B hemilineage, which contributes the 10c bundle [10] (n = 1/1, Figure 9A). These cells do not express *Ubx* (n = 2/2, Figure 9A’). *dronc*^*-*^ clones in the thoracic segments showed the additional 10i bundle, formed by the 10A siblings that normally die [10] (n = 1/1, Figure 9B), and in T3 such clones now had cells with moderate Ubx levels (n = 1/1, Figure 9B’). We recovered *dronc*^*-*^ clones in A1 (n = 2/2, Figure 9C), and these were strongly Ubx+ (n = 2/2, Figure 9C’). When we generated *Ubx*^*-*^ clones, we also found lineage 10 clones in A1, although these only contained the 10B hemilineage (n = 8/8, Figure 9D). With the ectopic expression of Ubx, we failed to find lineage 10 clones in any segment.

#### Lineage 11

Lineage 11 is normally present in T1 and T2, but not in T3 or in the abdomen [9]. Only the 11A hemilineage producing the 11im bundle is present in T1 (n = 3/3, Figure 9E), while 11id is also present in T2 (n = 7/7, Figure 9F), indicating the additional presence of the 11B hemilineage. Suppression of cell death resulted in the appearance of both hemilineages in T3 (n = 2/3, Figure 9I). Ubx expression in these neurons was weak in T2 (n = 5/6, Figure 9H’) but very strong in T3 (n = 3/3, Figure 9I’). The loss of *Ubx* also resulted in the appearance of lineage 11 clones in T3; however, only 11im was present, with 11id greatly reduced or absent (n = 11/13, Figure 9L). Interestingly, 11id was reduced or absent in T2 *Ubx*^*-*^ clones as well (n = 12/15, Figure 9K), indicating that a low level of Ubx is required for the survival of the 11B neurons that produce the 11id bundle. We found no lineage 11 clones when we ectopically expressed *Ubx* in the *UAS-Ubx* experiments.

Based on these data, *Ubx* is responsible for the lack of lineage 11 neurons in T3 and of lineage 10 neurons in A1. One possibility is that *Ubx* causes the death of both siblings right after they are born. However, in WT individuals, we have not seen any indications of these lineages in the respective segments, but we typically look at the end of larval life, so the NB and all of its progeny may have died by that time. Alternatively, Ubx expression may cause the death of the NB itself. Previously it was reported that postembryonic NB survival in the ventral CNS is governed by the mutually exclusive expression and antagonistic functions of *Antennapedia* and *Abd-A* but not *Ubx*[17]. The NBs that give rise to lineage 10 in A1 and to lineage 11 in T3 may be the exceptions to this rule.

### The ectopic expression of Ultrabithorax prevents survival of many Ubx negative lineages

Of the 25 postembryonic lineages, 13 lineages are without Ubx expression in WT clones (2, 4, 5, 10, 13, 14, 16, 17, 18, 20, 21, 22 and 24), and three express Ubx in just a few cells (8, 15 and 23) (Figure 2A). Because they are rarely labeled, we do not have Ubx misexpression data for lineages 5, 17 and 18. Within the remaining group of Ubx- lineages, lineages 2, 21 and 23 were unaffected by ectopic Ubx expression, and lineage 8 lost only the 8A hemilineage (Figure 2C). The remaining Ubx- lineages (4, 10, 13, 14, 15, 16, 20, 22 and 24), along with lineages 1, 11 and 12, all appeared to be killed by Ubx misexpression (Figure 2C). Of 375 *UAS-Ubx* clones, we recovered only one robust example of a lineage from this group. In their place we often found reduced cell clusters that appeared to be degenerating clones with greatly reduced cell numbers and/or axon bundles (Figures 3J,K and 8M,N,O; and not shown). By contrast, robust clones from these 12 lineages comprised 50% (n = 170/342) of WT clones, 47% (n = 194/416) of *dronc*^*-*^ clones, and 54% (n = 317/592) of *Ubx*^*-*^ clones. Therefore, these Ubx- lineages retain the ability to respond to Ubx even though they do not express it.

## Discussion

### Ultrabithorax expression patterns in the embryonic and postembryonic ventral nervous system

Metamorphosis in *Drosophila* brings about a profound change in body form. Although the thoracic and abdominal segments have relatively similar morphologies in the larval body plan, they then diverge dramatically in the adult body plan. Within the adult thorax, there are additional segmental specializations to accommodate segment-constant features (legs) and segment-variable features (wings and halteres). These segmental specializations are sculpted by the Hox genes, with Ubx being the major gene effecting differences within the thorax. The difference in the complexity of the body of the larva versus the adult is paralleled by a difference in the complexity of Ubx expression during embryonic and postembryonic development of the nervous system.

At hatching, Ubx expression is observed in most neurons in parasegments 5 and 6, with expression in the latter being the stronger [23] (Figure 1). This expression pattern appears to be stable in the embryonic-born neurons throughout larval growth. We have found Ubx expression in the lineages of adult-specific neurons to be quite heterogeneous. With the exception of anterior expression in the median lineage, and extended posterior expression in lineage 9, Ubx expression is still confined to the lineages in parasegments 5 and 6 (posterior T2 through anterior A1). Each lineage, though, develops as an autonomous unit, and each adopts a characteristic pattern of Ubx expression, with the postembryonic neurons in a cluster being either all Ubx+, all Ubx-, or mixed. In the case of mixed expression, Ubx expression is typically restricted to one hemilineage or the other, although it may be found in either the Notch^ON^ or Notch^OFF^ set of siblings (Figure 2).

For a given lineage or hemilineage, expression in parasegment 6 was higher than in parasegment 5 (Figures 3B,C, 4B,C, 5B,C, 6B,C, 8B,C and 9I,J). More extreme segmental differences in expression were seen for hemilineages 9A and 17A, in which there was no expression in parasegment 5 but strong expression in parasegment 6 (Figure 2). There were no cases of ‘flip-flopping”, in which Ubx was expressed in one hemilineage in parasegment 5 but in the other hemilineage in parasegment 6. In most cases, the different levels of expression we observed caused segment-specific differences in neuron survival and/or morphology (for example, hemilineages 1A, 6B, 12A and 11B).

The expression of Ubx was highly correlated with whether or not a given hemilineage contributed to a segment-constant (leg neuropil) versus a segment-variable (flight neuropil) portion of the CNS. Most of the hemilineages or lineages that contribute to the leg neuropils were Ubx- (3A, 4, 8, 9, 13, 14, 15, 16, 20, 21, 22 and 24), the major exception being hemilineage 1B, in which Ubx causes the death of inappropriate leg interneurons in segment A1. Hemilineages that contribute to the dorsal flight neuropil, by contrast, were typically Ubx+ (0A, 3B, 6, 7B, 11B, 12A and 19B).

### Multiple roles for *Ubx* in neuronal production and differentiation

In the postembryonic nervous system, positional information conferred by Ubx has dramatically different consequences depending on hemilineage. As might be inferred from the many cases in which neurons that are present in anterior thoracic segments are absent from T3 or A1, Ubx expression promotes programmed cell death in numerous hemilineages including 1A, 6B, 9A, 12A and 19A. By contrast, for hemilineages 19B and 11B, neurons are missing from an anterior segment (T1), and in these cases *Ubx* is required for hemilineage survival (Figures 5 and 9). Lineage 19 strikingly shows this dichotomy of context dependence since its B (Notch^OFF^) sibling requires Ubx for survival, whereas its A (Notch^ON^) sibling is killed by Ubx expression. Besides being involved in the selective death or survival of hemilineages, Ubx also regulates the segment-specific survival of whole lineages, as in the case of lineage 11 in T3 and lineage 10 in segment A1 (Figure 9), although, as discussed earlier, we cannot be certain whether this is executed at the level of the postmitotic neurons or the NBs themselves. While the latter two lineages make use of Ubx expression to remove lineages from inappropriate segments, many of the leg-related lineages have shut off Ubx expression to insure the survival of their neurons in the normal Ubx domain of expression. Therefore, we see that the ectopic expression of Ubx in these lineages results in their death, independent of segmental location (Figures 2, 3J,K and 8M-P).

We also see that Ubx expression can regulate segment-specific morphology without affecting cell survival. For example, the median lineage 0A neurons normally project to the pI commissure and elaborate their processes in T1 (Figure 6A), but they project past that point to the aI commissure in segments T2 to A1 (Figure 6B-D). These differences persist when cell death is blocked (Figure 6E-H). Therefore, the loss or gain of *Ubx* function is able to alter axon guidance and target recognition, presumably due to segment-inappropriate expression of signal transduction pathway components.

Besides lineage- and hemilineage-restricted patterns, we saw examples such as lineages 8, 15 and 23 in which *Ubx* expression was confined to only two to three cells in the cluster. Because our analysis was at the cluster level, we could not determine whether the loss of *Ubx* resulted in the death of this small number of cells. Also, the low-level *Ubx* expression in these cells might direct later events that occur as the neurons mature during metamorphosis.

We conclude that the effects of Ubx expression are not universal for secondary lineages but instead are lineage- and even hemilineage-dependent, implying independent co-option of *Ubx* by distinct mechanisms of regulation. This ability to act as a micromanager rather than a global switch would also allow *Ubx* to sculpt numerous species-specific differences in nervous system development during insect evolution without disruption of the largely conserved neuroblast array [23,24].

### Candidate mechanisms for context-dependent *Ubx* function

In the postembryonic CNS, *Ubx* carries out such diverse downstream functions as promoting NB or neuron death, promoting cell survival, and influencing axon guidance. These context-specific responses could be mediated through a number of different mechanisms: for example*,* expression levels governing threshold-dependent processes, alternative splicing, and the presence of specific cofactors and/or collaborators, any of which could influence DNA binding specificity and/or activation versus repression of gene targets.

Levels of *Ubx* expression are known to be important to developmental patterning. For example, in the *Drosophila* leg, gene dosage contributes to species-specific bristle patterns [25]. Moreover, low levels of Ubx are sufficient to repress an eighth bristle row on the posterior femur in T2 and T3, but higher levels are required for the repression of trichomes [26], suggesting that different levels of Ubx are required for distinct functions during development.

Similar differential responses to different levels of Ubx also appear to be in play for the postembryonic lineages since we see several cases in which neurons express Ubx at low levels in one segment but die in response to higher levels in the next. The best example is the 12A hemilineage, which makes the 12id and 12im axon bundles in T1 and the 12id (but not always 12im) bundle in T2, and dies in T3. These differences are associated with three levels of Ubx expression in this hemilineage: none in T1, intermediate levels in T2, and high levels in T3. Importantly, when the 12A neurons in T3 are rescued by *dronc* mutants, they produce only the 12id bundle, but when rescued by removal of *Ubx*, both the 12id and the 12im branches are produced. This suggests that low levels of Ubx prevent formation of the 12im branch, but high levels cause cell death.

The six Ubx isoforms are believed to have distinct roles in regulating target gene expression in different tissues during development [27,28], but it has long been standard practice to use a single isoform for overexpression studies. While our loss of function experiments were carried out using a null allele of *Ubx*, eliminating all possible isoforms, the gain of function experiments used a transgene constructed from the Ia isoform [29]. We nonetheless observed overexpression phenotypes that appeared to be the opposite of those from loss of function experiments, suggesting that Ia can substitute for the isoforms normally expressed in the CNS. This is consistent with reports that expressing any isoform ectopically at high enough levels can compensate for the lack of the normal one [30]. Nonetheless, it remains an untested possibility that different isoforms of Ubx are responsible for distinct functions in the postembryonic lineages.

A final possibility is that different Hox cofactors and/or collaborators are present in the cells of different NB lineages. Cofactors of the Pbx/Meis family (Extradenticle and Homothorax in *Drosophila*) are TALE homeodomain proteins that bind DNA cooperatively with Hox genes to increase target specificity *in vivo*, reviewed in [31]. The homeodomain protein Engrailed, which is differentially expressed between the siblings of at least some lineages (JWT, unpublished work), has also been shown to be a Hox cofactor and appears to be involved specifically in target gene repression via recruitment of the co-repressor Groucho [32,33]. In addition, other transcription factors such as Teashirt and Sloppy paired appear to function as Hox collaborators at Hox-targeted *cis* regulatory elements [32,34,35].

## Conclusions

We have found the Hox gene *Ubx* to be a key regulator of anteroposterior patterning in the postembryonic ventral nervous system of *Drosophila melanogaster*. In the larva, *Ubx* is not expressed homogenously within its general domain (parasegments 5 and 6), but rather in specific NB lineages and even hemilineages. *Ubx* is both necessary and sufficient for many of the segment-specific differences in NB lineage morphology observed in previous studies. Moreover, *Ubx* acts in a context-dependent manner, promoting programmed cell death, promoting cell survival, and/or regulating axon morphology, depending on the hemilineage. In some hemilineages, the function of Ubx is segment-specific and appears to depend on the level of expression. Thus, Ubx has been co-opted during evolution for multiple roles in sculpting the postembryonic ventral nervous system in a segment-appropriate manner.

## Methods

### Fly stocks

This study employed the MARCM (mosaic analysis with a repressible cell marker) system, in which the FLP/FRT method is used to generate clones of cells lacking GAL80, a suppressor of GAL4 [21]. This allows a reporter gene, *UAS-mCD8::GFP*, to be driven exclusively in cells that are homozygous mutant for a gene of interest or in cells expressing an additional UAS-transgene of interest.

The *w, GAL4*^*C155*^*, hsFLP, UAS-mCD8::GFP; FRT82B; tubP-GAL80* and *yw; FRT82B, Ubx*^*1*^*, e/TM6β* stocks, and the parent lines we used to generate a novel *yw; FRT82B, UAS-Ubx.Ia.C/TM6β* stock, were generous gifts from Jay Parrish and Yuh-Nung Jan (UCSF) [36]. The *p{ry+, neoFRT82B}, ry* stock used for WT MARCM was obtained from the Bloomington *Drosophila* Stock Center (Indiana University). Cell death was inhibited using flies of genotype *hs-flp, GAL4*^*C155*^*, UAS-mCD8::GFP/+; tubP-GAL80, FRT2A/dronc*^*ΔA8*^*, FRT2A*[37].

### Generation of MARCM clones

To generateWT control and *UAS-Ubx* MARCM clones, eggs of the appropriate genotype were collected for 12 h on grape agar plates at 25°C, incubated at 25°C for 12 h, and then heat-shocked at 37.5°C. Treatment was similar for *dronc*^*-*^ clones except that eggs were collected for 24 h and then heat-shocked immediately. To generate *Ubx*^*-*^ MARCM clones, eggs were collected for 12 h on grape agar plates at 25°C and then heat-shocked immediately. Following heat shock, embryos or larvae were transferred to instant fly media (Carolina Biological Supply, Burlington, NC, USA) and reared at 29°C to increase expression of the GAL4^C155^ driver and visibility of MARCM clones.

### Production of the anti-Ultrabithorax antiserum

To generate Ubx antibody 7701, we first expressed a fusion-antigen using the cDNA clone RE43738 received through the Drosophila Genomics Resource Center. The sequence chosen for its low complexity and low paralogy to other genomic regions was ATGAACTCGTACTTTGAACAGGCCTCCGGCTTTTATGGCCATCCGCACCAGGCCACCGGAATGGCAATGGGCAGCGGTGGCCACCACGACCAGACGGCCAGTGCAGCGGCGGCCGCGTACAGAGGATTCCCTCTCTCGCTGGGCATGAGTCCCTATGCCAACCACCATCTGCAGCGCACCACCCAGGACTCGCCCTACGATGCCAGCATCACGGCCGCCTGCAACAAGATATACGGCGATGGAGCCGGAGCCTACAAACAGGACTGCCTGAACATCAAGGCGGATGCGGTGAATGGCTACAAAGACATTTGGAACACG. The corresponding protein region is MNSYFEQASGFYGHPHQATGMAMGSGGHHDQTASAAAAAYRGFPLSLGMSPYANHHLQRTTQDSPYDASITAACNKIYGDGAGAYKQDCLNIKADAVNGYKDIWNT, which corresponds to position 1–106 of the Ubx protein.

**Figure 10:**
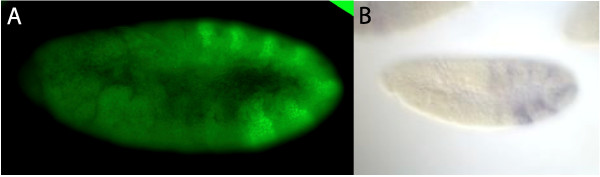
**Staining specificity of anti-Ubx_7701 antibody. **(**A**) *Drosophila y*; *cn bw sp* embryo at stage 11 stained with the anti-Ubx_7701 antibody showing specific expression of the protein compared with (**B**) the cDNA expression of the clone RE43738 (*Drosophila* Genomics Resource Center).

The complementary DNA sequence was cloned by LIC cloning within the pMCSG19 vector [38] to express and purify the antigen as described. The antigen was injected in rabbits by Millipore using their standard polyclonal protocol. Sera were then used to test antibody specificity by fluorescent immunostaining on *Drosophila* embryos (Figure 10).

### Immunohistochemistry

Nervous systems were dissected from wandering third instar larvae in PBS (pH 7.2), fixed in 3.7% buffered formaldehyde at room temperature, and then washed in 0.3% PBS-TX (PBS with 0.3% Triton-X100). Fixed samples were blocked in 2% normal donkey serum (Jackson Immunoresearch Laboratories, West Grove, PA, USA) in PBS-TX for 30 min and then incubated with rat anti-mCD8 (Caltag/Invitrogen, Grand Island, NY, USA) at 1:100 and either rabbit anti-Ubx at 1:1000 (7701) (Figure 10; [39]) or mouse monoclonal anti-neurotactin (BP106, Developmental Studies Hybridoma Bank, University of Iowa, Iowa City, IA, USA) at 1:30 for several days at 4°C. After primary antibodies were washed out, tissues were incubated overnight at 4°C in a 1:300 dilution of fluorescein isothiocyanate-conjugated donkey anti-rat IgG and Texas Red-conjugated donkey anti-mouse IgG (Jackson Immunoresearch Laboratories). After additional washes, tissues were mounted on poly-lysine coated coverslips, dehydrated through an ethanol series, cleared in xylene, and mounted in DPX (Sigma-Aldrich, St. Louis, MO, USA).

### Microscopy and image processing

Fluorescently stained nervous systems were imaged using either a 63× oil objective on a BioRad MRC 1024 confocal microscope or a 40× oil objective on a Leica SP5 Spectral Systems confocal microscope. Z-stacks were collected sequentially with averaging at 0.5 to 1.0 μm intervals.

Raw data stacks were imported into ImageJ (http://rsbweb.nih.gov/ij/) using a Bio-Formats plug-in (LOCI, University of Wisconsin, Madison, WI, USA) and either merged or projected into three-dimensional representations for analysis. Lineages were identified based on morphology, NB location, and/or projections into the neurotactin scaffold with reference to our published atlas [9,10].

Images are shown as single views of three-dimensionally reconstructed and rotated confocal stacks or as single optical slices, as indicated. Confocal stacks were processed and assembled into figures using ImageJ, Microsoft Powerpoint, and the Adobe Photoshop Suite. Multiple clones are typically labeled in the same sample and often obscure one another in a simple projection. For clarity, individual clones were cropped out in their entirety and adjusted for brightness and contrast. Only the slices featuring the most relevant portion of the neurotactin axon scaffold were projected and shown as landmarks.

For selected lineages, we made two-dimensional z-projections in ImageJ to measure the diameter of the axon bundle that exited the cell cluster. We used the *straight* line-drawing tool in combination with the plot profile macro to measure the diameter of the bundle as it crossed the midline.

## Abbreviations

A1: first abdominal segment; abd-A: abdominal-A; aI: anterior intermediate; aV: anterior ventral; CNS: central nervous system; GFP: green fluorescent protein; GMC: ganglion mother cell; Ig: immunoglobulin; MARCM: mosaic analysis with a repressible cell marker; NB: neuroblast; PBS: phosphate-buffered saline; PBS-TX: phosphate-buffered saline with Triton X-100; pD: posterior dorsal; pI: posterior intermediate; T1: first thoracic segment; T2: second thoracic segment; T3: third thoracic segment; Ubx: Ultrabithorax; WT: wild-type.

## Competing interests

The authors declare that they have no competing interests.

## Authors’ contributions

ECM conceived of the study, participated in the design and coordination, carried out the Ubx loss of function experiments, assisted with clonal analysis for all MARCM experiments, created figures, and drafted the manuscript. KED carried out and analyzed the Ubx gain of function experiments, assisted by ARC. DRA, KTR, and MEC carried out the wild-type and *dronc*^*-*^ Ubx expression experiments and assisted with data collection and analysis. NN and KPW generated and characterized the 7701 antibody to Ubx, created the relevant figure, and critiqued the manuscript. JWT participated in the design and coordination, carried out the early larval expression experiment, created figures, and helped to draft the manuscript. All authors read and approved the final manuscript.
